# Editorial to the Special Issue Optical Fiber Sensors in Radiation Environments

**DOI:** 10.3390/s23229117

**Published:** 2023-11-11

**Authors:** Flavio Esposito, Andrei Stancalie, Stefania Campopiano, Agostino Iadicicco

**Affiliations:** 1 Department of Engineering, University of Naples “Parthenope” Centro Direzionale Isola C4, 80143 Napoli, Italy; flavio.esposito@uniparthenope.it (F.E.); stefania.campopiano@uniparthenope.it (S.C.); 2 National Institute Laser, Plasma and Radiation Physics, Center for Advanced Laser Technologies (CETAL), RO-077125 Magurele, Romania; andrei.stancalie@inflpr.ro

## 1. Introduction

Optical fibers are well known for their use in high-speed data links and related sensors nowadays find application in different domains, such as structural health monitoring, distributed sensing, but also biological and chemical monitoring. They exhibit many benefits such as the high sensitivity and measurement resolution but mostly their immunity to electromagnetic interferences, multiplexing, and possibility to be employed in hazardous and constrained areas. In this context, the use of optical fibers and sensors in radiation exposed environments has gained huge attention from the scientific community, national agencies, and companies. Therefore, nuclear plants, high energy physics facilities, aerospace and medical domains are benefitting of the advantages of optical fibers with respect to electronic sensors as the use of latter is typically avoided when doses exceed a few Gy. Fiber optics are thus used, for example, for the development of radiation resistant data links or sensors able to operate under radiation fields, conversely, they can be also exploited as dosimeters by leveraging on the radiation induced effects upon materials. There are three main physical mechanisms which can occur when an optical fiber is exposed to radiation: the radiation induced attenuation (RIA), consisting of an increase in the propagation attenuation; the radiation induced refractive index change (RIRIC), as glass structure can be modified leading to silica compaction and refractive index variations; radiation induced emission (RIE), as light can be generated into the fiber core and reach the detector based on different mechanisms (e.g., Cherenkov effect and luminescence) [1,2,3,4,5,6,7,8,9,10,11].

The Special Issue “Optical Fiber Sensors in Radiation Environments” of *Sensors* collects fourteen high-quality papers: one is a review article and the remaining are original articles. Two main aspects were addressed by their authors, which are the assessment of optical fibers and sensors under different kinds of radiations and the development of dosimeters. The works focused both on novel fiber configurations and metrological evaluation of commercial devices.

## 2. Overview of Contributions

A leading topic is the assessment of the performance of commercially available and unconventional optical fibers and fabricated sensors under radiation fields. 

Morana et al. (contribution 1) focused on a commercial ultra-low loss single-mode pure silica core fiber, i.e., model Vascade EX1000 from Corning. Nowadays, pure silica and F-doped core fibers are the most widely used when radiation hardness is required. Differently, high RIA levels of about 3000 dB/km at 1310 nm and 2000 dB/km at 1550 nm at 2 kGy dose X-rays were measured for the fiber under investigation. Moreover, most of the RIA was recovered after the irradiation, due to the metastable nature of the radiation induced point defects causing the fiber degradation. The authors associated the unexpected behavior to the specific manufacturing process of the fiber employed to reduce the propagation attenuation, hypothetically the presence of alkali metals. As a consequence, the authors point out that not all pure silica core fibers are well suited for radiation resistant applications and propose the use of this particular fiber for beam monitoring. 

In our work (contribution 2), we have reported about a new setup for the gamma irradiation of optical fiber components and sensors based on a compact Co-60 gamma chamber, model GC-5000 (G.C., India). The irradiator exhibits a 5000 cm^3^ space where the devices can be exposed to a homogenous gamma flux due to cylindrically arranged Co-60 bars. Fiber patchcords allow the connection to outside environment, permitting the real-time measurement of the devices under irradiation. As a study case, the results of the gamma irradiation of long period gratings (LPG) are presented and compared to those achieved into an industrial irradiator, showcasing better control of the irradiation conditions and easy operation for the newly proposed setup. 

The same setup was also employed in the work with Theodosiou et al. (contribution 3) for the gamma irradiation of femtosecond laser written fiber Bragg gratings (FBG) using the point-by-point (PbP) and plane-by-plane (PlbPl) methods into a standard single-mode fiber. In the same work, the electron irradiation of similar set of gratings was also performed up to the same total dose of 15 kGy. The authors compared the FBG spectra before and after irradiation as well as their temperature sensitivity. They found a stronger effect induced by electrons with respect to the gamma irradiation for both fabrication methods, moreover the FBGs written by PlbPl method demonstrated higher radiation resistance than those achieved by PbP. 

FBGs were also investigated by Lebel-Cormier et al. (contribution 4) as dosimeters for medical applications. They embedded the gratings into different kind of polymers and found noticeable response down to 0.3 Gy with a maximum sensitivity of 0.087 pm/Gy, based on the radiation induced thermal expansion in the polymer coating. The authors supported the experimental findings with a simple physical model based on the thermal and mechanical properties of the surrounding polymer. This configuration shows potential for radiotherapy application with MRI-linac apparatus. 

With the aim of better understanding the effect of radiations on optical fiber sensor and components, Rana et al. (contribution 5) numerically investigated the macroscopic effects of refractive index change on three resonance based devices, such as FBG, LPG and Fabry-Perot cavity. Specifically, starting from the radiation induced refractive index values from literature, the authors used the Lorentz–Lorenz relation to calculate the corresponding density and length change in the optical fiber and subsequent effects on sensor response.

Optical fiber is a valuable mean for the monitoring of radiation exposed environments, as for example in the work of Lee at al. (contribution 6) where they proposed a passive fiber optic network for the remote monitoring (more than tens of km) of water level in a spent fuel pool inside a nuclear power plant. The working principle is based on the change of Fresnel reflection power coefficient at the sensing points in real-time.

Finally, Rovera et al. (contribution 7) presented a comprehensive review regarding the use of fiber optic sensors for aerospace applications, as such environments are exposed to radiations and harsh conditions and benefit of the advantages of fiber optic technology. The work focused on the main aerospace requirements, the working principle and radiation damages of optical fibers and related sensors. Moreover, they showed various examples of applications in radiation exposed environments related to aerospace. The authors highlight that, currently, is still necessary to measure the response to radiation of each kind of sensor as it is not simple to predict their behavior due to the complexity of the mechanisms involved. Packing should be also addressed to protect them and enhance their lifetime.

Another important issue is the development of radiation sensors or dosimeters. Söderström et al. (contribution 8) studied the radiation induced emission of sol-gel silica rods doped with Ce-, Cu-, and Gd- ions, under a pulsed 20 MeV electron beam. These samples were connected to a multi-mode pure silica core fiber for the readout of luminescence signal. Investigations were conducted to a pulsed electron Clinac beam in use for radiotherapy, for the first time. The authors investigated the luminescence pulses in the samples induced by the electron bunches with respect to deposited dose per electron bunch. A linear response in terms of luminescence depending on the electron bunch sizes, ranging 10^−5^–1.5×10^−2^ Gy/bunch, was obtained. Such investigation demonstrated that those materials could find application for the radiation monitoring of electron Clinac beams. 

Cieslikiewicz-Bouet et al. (contribution 9) proposed the incorporation of Ce^3+^ ions into pure silica matrices by using the modified chemical vapor deposition (MCVD) technique for the development of luminescence-based sensors. MCVD is the reference method for the fabrication of optical fibers with better control of the glass composition and was applied to Ce-doped pure silica glasses as a novelty. The structural and optical characteristics of the fabricated samples were studied by means of different spectroscopic techniques. The corresponding drawn fiber was exploited for remote X-ray radiation dosimetry, exhibiting a radioluminescence signal with a linear behavior versus dose rate over five decades (330 µGy/s–22.6 Gy/s range). 

Shin et al. (contribution 10) reported about a flexible in-vivo dosimeter based on a copper indium gallium selenide (CIGS) solar cell for use under therapeutic X-ray radiation. The metrological assessment involved the basic features, such as dose linearity, dose rate independence, energy independence, and field size output. Dose linearity/dose rate independence was observed with the possibility to adapt the size and shape of the cell for the specific application. 

Wolfenden et al. (contribution 11) developed a novel machine protection system for high power particle accelerators based on optical fiber technology. The working principle is based on the generation of Cherenkov radiation when energetic charged particles pass through the sensing fiber. The same fiber also transmits the Cherenkov pulse to the detector and location of the source can be performed through time-of-flight measurements. The system proved successful for the detection of both beam loss and radiofrequency breakdown, which are among the main failures occurring in this scenario, and displays advantages in terms of spatial resolution and covered length.

Extensive research and commercial products are focused on scintillating materials. In the work of Devic et al. (contribution 12), a metrological assessment of the performance of the commercial real-time dosimeter IVISCAN from Fibermetrix for computed tomography (CT) is reported. It is based on a plastic scintillating optical fiber and the authors evaluated the performance and uncertainty regarding the dependence upon the dose-rate, angle, cumulative dose, energy, length, as well as the repeatability and spatial uniformity, in reference and clinical CT beam qualities. The results highlighted that the performance of the system meets the international standard IEC61674 related to X-ray diagnostic imaging. 

Jelinek et al. (contribution 13) proposed a gamma dosimeter consisting of a scintillating crystal, a connecting fiber and a scintillation detector. They tested different solutions for each of the three previously mentioned parts of the system. The best configuration uses a LYSO crystal, a 1.5 mm diameter silica multi-mode fiber, and a photomultiplier. The system has interchangeable sensors, fibers and detectors permitting to be adapted for the specific application up to hundreds of kBq. 

Thrower at al. (contribution 14) evaluated the performance of the commercially available plastic scintillator detector Exradin W2 by Standard Imaging (USA) in a 10 MV flattening-filter-free (FFF) photon beam. It is found that such scintillator is ideal for small field dosimetry of high dose rate beams. 

## 3. Conclusions

This Special Issue focused on the latest developments and trends in optical fiber sensors for radiation environments, covering recent improvements in the related theory, design, fabrication, and application/validation. The high-quality papers provided a useful insight of the present status and future outlook in this area. The attention of the authors of the accepted manuscripts was mainly focused on the evaluation of optical fibers (commercial and unconventional ones) and sensors (FBG, LPG, etc.) when exposed to different types of radiations. Moreover, huge attention was focused on dosimeters (e.g., based on scintillators) in high energy applications and medical field. Few papers also evaluated the performance of commercial devices in order to foster their application in such domain.
